# ‘It’s just a great muddle when it comes to food’: a qualitative exploration of patient decision-making around diet and gout

**DOI:** 10.1093/rap/rkab055

**Published:** 2021-08-13

**Authors:** Jennifer Liddle, Jane C Richardson, Samantha L Hider, Christian D Mallen, Lorraine Watson, Priyanka Chandratre, Edward Roddy

**Affiliations:** Population Health Sciences Institute, Newcastle University, Newcastle upon Tyne; School of Law and Social Justice, University of Liverpool, Liverpool; School of Medicine, Keele University, Keele; Haywood Academic Rheumatology Centre, Midlands Partnership NHS Foundation Trust, Burslem; School of Medicine, Keele University, Keele; School of Medicine, Keele University, Keele; Sandwell and West Birmingham Hospitals NHS Trust, Birmingham, UK; School of Medicine, Keele University, Keele; Haywood Academic Rheumatology Centre, Midlands Partnership NHS Foundation Trust, Burslem

**Keywords:** gout, inflammatory arthritis, diet, qualitative, patient experience, self-management, long-term condition

## Abstract

**Objective:**

Our aim was to understand whether, why and how patients choose to modify their diets after developing gout.

**Methods:**

We conducted an inductive thematic secondary analysis of qualitative data from 43 interviews and four focus groups with UK participants with gout (*n* = 61).

**Results:**

Participants commonly initiated dietary changes as part of a self-management strategy for gout. Reasons for making such dietary changes included: desperation; a desire for control; and belief that it would be possible to achieve successful management through diet alone; but not weight loss. Participants who did not make changes or who reverted to previous dietary patterns did so because: they believed urate-lowering therapy was successfully managing their gout; medication allowed normal eating; they did not find ‘proof’ that diet would be an effective treatment; or the dietary advice they found was unrealistic, unmanageable or irrelevant. Dietary modification was patient led, but patients would have preferred the support of a health-care professional. Beliefs that diet could potentially explain and modify the timing of flares gave patients a sense of control over the condition. However, the belief that gout could be controlled through dietary modification appeared to be a barrier to acceptance of management with urate-lowering therapy.

**Conclusions:**

Perceptions about gout and diet play a large role in the way patients make decisions about how to manage gout in their everyday lives. Addressing the reasons why patients explore dietary solutions, promoting the value of urate-lowering therapy and weight loss and drawing on strong evidence to communicate clearly will be crucial in improving long-term clinical management and patient experience.

Key messages

- Desperation and desire for control can lead to patients initiating dietary changes after gout diagnosis.
- Beliefs that gout can be managed by diet alone can reduce urate-lowering therapy uptake.
- Patients usually self-manage dietary change but would prefer support from health-care professionals.

## Introduction

Gout has been synonymous with diet and lifestyle in popular culture for centuries [1–4]. It is the most common inflammatory arthritis, affecting 2.5% of UK adults [5]. Inherited risk factors, older age, co-morbidity, male sex and ethnicity are key risk factors for hyperuricaemia and development of gout [6]. However, it is only recently that the associations between dietary factors and gout incidence have been confirmed in large epidemiological studies [7]. Higher levels of consumption of meat, seafood, sugar-sweetened soft drinks, fructose, alcohol (particularly beer) and Western dietary patterns (higher intake of red and processed meats, sugar-sweetened beverages, sweets, desserts, French fries and refined grains) are associated with hyperuricaemia and an increased risk of developing gout, whereas dairy products, coffee, vitamin C and Dietary Approaches to Stop Hypertension dietary patterns are associated with reduced risk [8–14].

Although renal and gut excretion of urate is central to regulation of serum urate [6], people commonly attribute gout flares to the consumption of specific food types, based on widely held perceptions that dietary factors trigger flares [15–17]. There is evidence that consumption of animal purines, cherries, omega‐3 polyunsaturated fatty acid-rich fish and alcohol might affect the risk of flares occurring in people with gout [18–22]. However, despite the interest of people with gout in dietary intervention [23–25], there is little evidence of the clinical effectiveness of dietary interventions to lower serum urate, prevent flares or reduce the volume of monosodium urate crystal deposits [26, 27]. Furthermore, patients’ beliefs about which dietary factors are important may not accord with the existing evidence. For example, one survey found that more patients believed that vegetables triggered flares than believed that beer did [28]. In contrast, there is strong evidence demonstrating that urate-lowering therapy (ULT) is highly effective in reducing flares [29] by reducing serum urate to a concentration that achieves dissolution of monosodium urate crystals. However, patients’ interest in, and beliefs about, dietary intervention as an effective alternative to long-term medication might be a barrier to successful uptake and adherence to ULT, although more evidence is needed [25, 30, 31]. The aim of this study was to understand whether, why and how patients choose to modify their diets after developing gout.

## Methods

### Design

This study was an inductive thematic secondary analysis of qualitative data from interviews and focus groups with UK patients with gout. Our methodological approach draws on interpretative phenomenological analysis theoretical frameworks [32, 33]. We focus on individual lived experiences of gout and seek to describe their meaning in terms of both what was experienced and how it was experienced.

The aim of the two original studies was to explore participant experiences of gout [34, 35], rather than to investigate views on diet. However, participants in both studies regularly raised the topic, leading to this secondary analysis. The original research received ethical approval (NRES Committee South Central Berkshire 12/SC/0495 and 09/H0505/66; North West Liverpool East Local Research Ethics Committee 12/NW/0297). Written informed consent to participate and use data in future analyses was obtained from all participants.

### Data collection and participants

A dataset comprising anonymized transcripts was drawn together from two previous qualitative studies. One study comprised one-to-one semi-structured interviews with 43 people with gout (14 women and 29 men; age range 32–87 years; time since diagnosis ranging from 1 to ≥16 years; flares in past 12 months ranging from 0 to ≥10) [34]. Of these participants, 24 (8 women and 16 men) were currently using ULT, and 5 (2 women and 3 men) had used it in the past but had stopped. Recruitment and data collection was carried out by J.L. (female, Research Associate experienced in qualitative research, not involved in medical care of the participants). Interview participants were recruited from UK general practices, rheumatology clinics, gout support groups and online advertising. The other study involved four focus groups (2 women and 16 men; age range 55–85 years) [35]. Seventeen focus group participants had gout and were sampled from participants in a primary care cohort study (flares in past 12 months: range one to five) [36]. One female participant was a relative (carer) of another participant but did not have gout herself. Ten participants (one woman and and men) were using ULT. Focus group recruitment and facilitation was conducted by P.C. (female, PhD student, specialist registrar in rheumatology, not involved in medical care of participants). Recruitment, participant characteristics and data collection have been reported previously [34, 35].

### Analysis

J.L. read and re-read all anonymized transcripts to become immersed and familiar with the data. Analysis software (NVivo 12, QSR International (UK) Limited, Daresbury, Cheshire, UK) was used to support data management and retrieval. Following the principles of thematic analysis, J.L. completed an inductive line-by-line coding of all data related to gout and diet [37], collating a list of all codes and data extracts across the transcripts. Codes were condensed to eight focused (superordinate) themes (see Table 1), representing clusters of themes capturing the experiences of participants [33, 37]. The themes and list of codes were shared with a second researcher (J.C.R.), and interpretations of the data and participant experiences, attitudes and beliefs were discussed.

**Table 1:** Organization of themes relating to gout and diet

Superordinate themes	Example data
Desperation and desire for control over condition and its management	When you’ve got gout, you will try anything, and I mean anything, and I’ll eat cherries, I’ve had baking powder, I’ve had cider vinegar, er, honey, er, that type of thing; they’ve only got to mention it on the Internet that it might help, then you try it, you will try it, honestly, and, for the pain. And, er, you know, the baking powder I have it every day for quite a while, I could never prove that it helped, I could never prove that it did me any harm, er, it might have helped, I don’t know, but, er, if you think it’s doing you good, like cider vinegar, you will take it and, er, you might convince yourself and the gout that it’s working [laughs]. (William) My doctor is lovely. He does not demand. […] He’s very open to talking to you about it, and he accepted and allowed me to do—to go down the path I wanted, which was to try and avoid medication and, as I say, watch my diet. He allowed me to do what I was comfortable with. (Gail)
Perception that successful management of gout through (individualized) dietary modification is possible	I’ve gone nearly two-and-a-half years without an attack. So, when I had my last one I was really surprised I’d got it, but then thinking back, I’d introduced another foodstuff that I’ve never took and now I’m aware of it. I’m really thinking, ‘I’m going to spend the rest of my time and never get it’. I’m very positive about that—never get an attack of gout again. […] I think the best thing would be […] not to just say, ‘Well, I’ve got gout; the tablets will sort it out’. Without actually trying to say, ‘Well, what’s actually causing it in the first place?’ Really, if you look at it, I think for the majority of people, if you could find out what’s caused it in the first place then it probably will prevent you from having gout, or if not prevent, then reduce the number of attacks you have. I’m sure all of us have got some trigger factors. That would be my advice, to be sort of—to attack the disease and not just accept it. (Graham)
Weight loss not a central aim of initial dietary modifications	And, of course, there had been a change in my routine, which was that I stopped taking serious exercise, and […] I put on weight and all the rest of it. And I, I now discovered, through talking to one or two other people, I’ve only got a small sample, but this was their experience as well. Having done jobs—a gamekeeper, for instance, who used to walk, you know, miles and miles every day and then suddenly he retired and he had gout, almost within weeks. […] I’ve now got reasonably fit over the last few months and I’ve lost a bit of weight, although you’ve really got to do serious things to lose weight seriously and change your diet and everything. (Male focus group participant) Because I think deep down, it’s started developing maybe the issue when I was heavy […] so I know full well I’m going to have to keep—keep my weight down definitely, and watch my diet. I can’t sway […] you’ve always got to be mindful of eating and drinking the right thing, I think that’s the important thing. (Georgina) I’m now over 20 stone, don’t exercise, can’t exercise, other conditions. I don’t particularly cut any food out. I don’t know what caused the gout to come. I’ve—nothing I’ve done different in the last 15, 20 years before the gout came. (Male focus group participant)
Experimenting and following self-imposed dietary rules or restrictions	The other day I was checking on the Internet and there was somebody who said ginger ale helps, so I went down to Waitrose and bought 3 litres of ginger ale and I’ve been drinking that ever since [laughs], but actually the gout started to go, whether it was going on its own accord or because of ginger ale I don’t know. But that’s the sort of thing I do, yes. (William) The trigger foods are just hard to pin down, for me, anyway, you know. And I don’t think there’s a definitive list. I don’t think there’s an actual top 10 list that these are the worst things. It’s, it’s, it’s kind of a process of, ‘I’ve had an attack. Okay, what did I eat in the last 2 days that might have triggered it? Is it shellfish? Is it…?’ If it’s you’ve had a lot of beer, the night before or whatever, you know. I don’t know. (Andrew) One thing I don’t eat anymore is oranges […] or lemons or grapefruit, I’ve packed them in […] I’m not chancing it […] it’s in my head that they might do something, just the thought of “might” will do for me– I’m not doing it. (Steve) You can get from herbal shops—cherry, you know, tablets based on those kinds of fruits and whatnot. I took those for a while to see if those would help at all. (Henry) We bought, we, myself and my partner, we got, you can get gout recipe diet books, so, so foods that are low in purines. (Adam)
Successful management with urate-lowering therapy removes perceived need for dietary modification	Interviewer : And have you tried any other remedies at all, or did you try anything else other than…? Paul: I’d no need to, that [allopurinol] was the remedy for me, and it works. Male focus group participant A: Well I’m still eating mussels and king prawns and everything like that. Male focus group participant B: The allopurinol, I suppose, is to let you do that isn’t it?
Dietary modifications perceived as unrealistic, unmanageable, unproven in effectiveness, or irrelevant	I’ve tried, oh, all sorts of herbal remedies […] I’ve tried literally dozens of them; they don’t work. They don’t work. They’re all expensive. And I think if anything they just make you feel sick. So … it—they didn’t work for me, the alternative medicines. And I tried dieting, and that didn’t work either. It didn’t seem to have any effect at all, what I ate. […] My view was that if they work, all the GPs would know that they work, and they would prescribe them for you. And so, I thought, waste of my time here. (George) I have looked up on the Internet, obviously, the causes of gout, but basically, it can be caused by practically everything you eat. You know, it’s either stop eating [laughter] and, hopefully, your gout will go away, or I don’t know. (Dorothy) The diet sheet said, […] these are the things to try and avoid, and it was things like herrings, tomatoes, anything acidic, and as I don’t like anything acidic anyway it’s not really a problem because I don’t eat acidic foods or drink acidic drinks, because I don’t like them. […] It wasn’t really of much use, was it, in the end? (Sandra)
Contradicting and confusing information	I think the gout, because it’s […] a bit like mercury. It’s very hard to pin down exactly, and everyone’s different. So, if it was a definitive—if it was more definitive, and said, ‘If you have gout you should not drink, or do not drink, or, you know, in—research has shown that, you know, in 80% of people who stop drinking, their gout went away’. If there was more definitive facts and figures like that, I think it would be easier for people to make life choices and make decisions, but I think it’s—because it’s, it’s vague, you know, understandably, it’s vague for various reasons, that people can take out of it what they want, you know, and it’s open to interpretation. (Andrew)
Dietary modification as a patient-led process	So, I think with my doctor it’s sort of looking to give me a painkiller, they don’t sort of give you the information that you can get on the Internet telling you what food can bring on gout and things like that, and that’s what I find has helped me a lot, going onto the Internet and understanding it more, and if you understand it, it’s less fearful. […] That’s when I learned more than ever, more than the doctor could ever tell me, and it was the diet that I started with, going on the Internet, yeah, it was that, that started it all. My doctor’s a superb doctor but, as he said, he’s got very little knowledge of gout and […] all they want to do is try and relieve you of the pain, not the cause, and that’s what I did find out yes. (William) You’ve got to do it yourself. (Female focus group participant) The gout websites […], they are a good support mechanism for people who use them. […] It’s good to be able to read about it and just see other people’s experiences. […] It’s a story, isn’t it? People can relate to it more and […] just understand it more if it’s coming from the horse’s mouth. (Andrew) My dad’s friend who’s been diagnosed with gout, he swore by cherry juice, so I tend to have a couple of glasses of that during the week. (Georgina)

## Results

Analysis resulted in eight superordinate themes: desperation and desire for control (T1); perception that successful management of gout through dietary modification is possible (T2); weight loss not a central aim (T3); experimenting and following self-imposed dietary rules (T4); successful management with ULT removes perceived need for dietary modification (T5); dietary modifications perceived as unrealistic, unmanageable, unproven or irrelevant (T6); contradicting and confusing information (T7); and dietary modification as a patient-led process (T8). Table 1 provides an overview of the themes and example supporting data. Each theme is then discussed in detail in the main text, using occasional illustrative quotes from participants. We have used the terms flare and urate in the main text, as recommended in the Gout, Hyperuricaemia, and Crystal-Associated Disease Network consensus statement [38]. However, in order to maintain authenticity in our textual representation of participants’ speech, alternatives, such as attack and uric acid, are retained where participants are quoted directly. All interview participant names are pseudonyms. Focus group transcripts did not track and identify individual speakers.

Four types of behaviour in relationship to diet were identified: (1) no dietary changes; (2) modification of diet followed by return to previous habits; (3) making and maintaining dietary changes; and (4) continued experimentation with diet.

### Why participants modified their diet after diagnosis

The intensity and frequency of flares often led to feelings of desperation and willingness to ‘try anything’ (T1). Beliefs that dietary intake could potentially explain and/or modify the timing and frequency of flares (T2) (rather than dietary factors predisposing to hyperuricaemia and intervention lowering urate) were central to participants’ motivations for dietary modification. For some participants, the consequences of these beliefs were long periods of time spent trying to identify the cause of their flares and living in fear of inadvertently consuming something that would induce a flare.

Beliefs about gout and diet afforded other participants a sense of control (T1) over what was seen as an unpredictable condition; also an important factor for those who wanted to avoid medication, had difficulty swallowing tablets or where safety of medication was uncertain. Some participants were searching for ‘a cure’, suggesting that they did not see medication to manage the condition as sufficient. However, some participants reflected that their hopefulness about the possibility of preventing gout flares with dietary changes (T2) had been a barrier to accepting management using ULT at an earlier point:

Looking back at my own story, and particularly reading things like the [online gout forum], I think there’s a recurring pattern in people’s stories. So, people get diagnosed. Try to rationalize what’s going on. What caused it? Is it something I’m doing? Can I do something differently? And end up on, invariably end up on allopurinol or febuxostat […] I think a lot of people try to find these natural cures and remedies. Some people rely on those and don’t want to go on a drug, but actually, life on the drug is a lot simpler than, you know, having a, you know, bizarre limitations on your diet, which don’t really have any impact. (Adam)

I suppose, because of the way I am, I, I am so anti taking things that probably it was a case of me coming round to accepting that I didn’t have any other options. But, as I say, it’s always easier to be wiser after the event. But if there was anybody, as I say, like me, […] then what I would say is obviously do, do what you feel best doing, but maybe if you find, as I say, that you’re getting frequent attacks, don’t perhaps be quite so adamant as I was to just carry on down that sort of, ‘Oh, I’ll watch what I’m eating’, because maybe the medication would have been a better option a bit sooner in my, my experience. (Gail)

Underpinning evidence supporting such beliefs came from widespread claims about the individualized nature and existence of ‘trigger foods’ that could induce flares, and anecdotal success stories of others with gout. Participants also made connections between their flares and their individual consumption of particular items in the preceding days. Perceived emphasis on the role of diet in gout management by general practitioners (GPs) was a confirmatory factor in the decision-making process. In some cases this was explicit, and GPs had attributed a recent flare to particular food patients had eaten. However, in other cases participants interpreted their GP’s questioning about their dietary habits as suggesting that diet must play an important role in the condition.

Participants were often aware of the connections between gout and being overweight, but had not made any dietary changes themselves with the aim of losing weight specifically to manage their gout (T3). However, in hindsight, Georgina believed that her weight might have contributed to development of the condition and hoped to maintain a healthy weight in the future. In contrast, some participants did not seem aware of the potential role of weight in gout. For example, one participant described how he weighed >20 stone (>127 kg), did not exercise and did not know what had caused his gout flares. He appeared to make no connection between these factors.

### How participants changed their diet after diagnosis

Participants described a range of approaches, including experimenting and looking for patterns systematically or by ‘trial and error’, aiming for ‘moderation’, and following advice or information from other sources (T4). Adam bought a serum urate monitor, which he calibrated and used to look for relationships between his measurements and what he ate. Georgina had a family friend with gout, who was adamant that cherry juice was beneficial; therefore, she had started drinking it regularly herself. Other participants believed they could link flares to items that they had eaten in the previous days: ‘If I see […] a cake, say a cream doughnut or something […] and I’ll say, “Oh, I’ll have one”, within 5 days I’ll have an attack of gout’ (male focus group participant).

Moderation was a concept with different meanings for each individual. George described his consumption of two pints of beer per week as ‘virtually teetotal’. A male focus group participant limited drinking Guinness to once per month. Steve drank beer only at weekends, whereas Colin had reduced his wine consumption from up to two bottles to half or one bottle per night. Margaret ate shellfish regularly but not more than twice per week.

Participants reported modifying their diets by adding, increasing, removing or reducing particular foods and drinks and/or introducing supplements and herbal remedies. Examples of these items are listed in Table 2.

**Table 2:** Examples of dietary changes made by participants

Items participants reported reducing or removing from their diets	Beer Brandy Cava Champagne Guinness Port Red wine Whisky Fizzy drinks Fruit juice Grapefruit Lemon Orange Pineapple	Asparagus Baked beans Broccoli Broad beans Butter beans Cauliflower Chickpeas Mushrooms Peas Spinach Tomatoes Herrings Mackerel Mussels Oily fish Prawns Scallops Squid	Beef Game Liver Lamb Red meat Strong cheeses Strong curries Marmite Yeast Protein

Items participants reported introducing or increasing in their diets	Coffee Ginger ale Water Cherry juice Cherry concentrate Cherries Beetroot Celery Spinach	Baking powder Honey Devil’s claw Epsom salts Milk thistle Vitamin C	

Perceptions about the purine content of specific foods and the benefit of increasing fluid intake influenced the choices participants made. Some participants believed that ‘acidic’ foods should be avoided and had stopped eating certain fruits and vegetables that they believed were too acidic and would cause flares. This confusion perhaps derived from information that participants had found or received about the role of urate (commonly described as uric acid) in gout. Urate and purines were also conflated, with participants describing certain foods being ‘high in uric acid’.

### Why participants did not (continue to) modify their diet after diagnosis

Participants who had positive experiences of ULT and believed this was controlling their gout successfully were less likely to report trying or continuing with dietary modifications (T5). The perception that medication allowed normal eating was seen as a key benefit alongside the absence of gout flares. Steve reported how ‘pleased’ he was because he had not had a flare for 3 or 4 years but had not modified his diet.

Lack of ‘proof’ or belief in the effectiveness of dietary modifications also contributed to participants ceasing, or not attempting, dietary change. Participants sometimes reported frustration or disappointment at noticing no effect from changes they had made, or expressed uncertainty about whether any differences they noticed (e.g. more or less frequent flares) were down to coincidence or attributable to what they had/had not eaten. Some participants had drawn conclusions about the (lack of) effectiveness of dietary modification as a management strategy from conversations with their GPs.

The perception that dietary modifications were unrealistic, unmanageable or irrelevant was another common concept (T6). Components of this included: the quantity of (and conflicting information about) foods purportedly impacting on gout; perceptions of contradictions between dietary advice for gout and for other health conditions; impacts on family/social life (such as social situations based around drinking beer, impacts on those who did the cooking, and eating together as a family or when out with friends); adverse consequences; and beliefs that current consumption patterns already matched those advised for people living with gout.

### Choosing and using information

The Internet was a primary source of material about gout and diet for participants, who valued its provision of detailed information that they felt was not easy to obtain from their GPs. Participants perceived GPs as having insufficient resources to support adequate acquisition of information and knowledge about dietary influences. They reflected that being guided by someone with accurate knowledge about the topic of diet, once they were diagnosed with gout, would have made a positive difference to them.

There was also a strong view that the onus was on individual patients to seek information about gout and diet, rather than this being a topic that GPs proactively raised or elucidated (T8). Personal success stories of others with gout were regarded as important evidence supporting, or stimulating ideas about, dietary modifications to try next. Participants were often comfortable basing their decisions on anecdotal evidence, and also continued to follow the dietary rules they had created over time even when they could not recall the original reason, inspiration or accuracy of information behind their belief that a specific modification might be effective. However, there was a perception by some participants that information sources were contradictory and confusing (T7), with a desire expressed for ‘official’ or factual online information.

## Discussion

The aim of this study was to understand more about whether, why and how patients choose to modify their diets after developing gout, increasing our understanding of the rationale behind patients’ choices. Dietary changes were commonly considered after diagnosis. Whether changes were made and continued, or not, depended on levels of desperation and beliefs about the potential role of diet in preventing flares. When ULT was commenced soon after diagnosis and subsequently reduced or prevented flares, participants saw little need for dietary intervention. However, when participants were not offered ULT, were resistant to the idea of long-term medication or experienced ongoing painful flares, their attempts to gain control and understanding of the symptoms led them to search for dietary causes.

Dietary modification was a patient-led process. Some participants viewed doctors as a source of medication information and provision, but not dietary advice. The ability to ‘do it yourself’ when it came to researching dietary factors was not a preference, but participants perceived there to be no realistic alternatives. Studies suggest that patients typically receive little education from GPs about the associations between gout and diet [28, 39–43]. Gout is not alone as a diagnosis that is rarely accompanied by evidence-based professional advice about diet, yet often prompts dietary change by patients [44]. Our study participants reported finding it difficult to obtain information from their GPs about dietary influences on gout. In some cases, GPs’ remarks about links between gout and diet had strengthened participant beliefs in the role of diet in managing gout. The Internet was the main information source for most people. However, online information about gout is often poor and does not convey key concepts about gout and its treatment or about the ethnic and gender diversity of people with gout [2, 23, 24, 45, 46]. Dietary advice may not be prioritized in the limited time GPs have available to talk to patients, but our study shows that patient perceptions about the role of diet can have longstanding impacts on how well their condition is managed. Web-based platforms might also be useful in addressing ongoing educational needs of gout patients [47].

Value was often placed on personal stories and anecdotal evidence. Others with gout urged continued experimentation based on the supposed individualized nature of ‘trigger’ foods and related difficulty in identifying them. This evidence was often central to participants rationalizing and maintaining their commitments to pursuing dietary solutions, despite no improvements in their symptoms. They concluded that they had yet to find/eliminate the particular food that would work for them specifically. Unrealistic expectations about individualized dietary solutions might be a barrier to uptake of ULT, leading to patients living with avoidable flares and long-term joint damage, and providing an opportunity for intervention.

Interestingly, participants’ interest in diet and dietary modification was almost exclusively focused on which dietary factors trigger gout flares and could be avoided to prevent/reduce flares. Gout was often considered an acute episodic rather than a long-term condition [17]. There was little appreciation of the possible urate-lowering effects of dietary modification. Similar knowledge gaps among nurses and patients’ partners have been described recently [48]. Participants were mostly unware of the contribution of being overweight/obese to hyperuricaemia and did not cite weight loss as a motivation for dietary modification. The population attributable risk of hyperuricaemia is higher for being overweight or obese (44%) than for specific dietary risk factors [49]. However, most participants could not recall being advised about the benefits of losing weight or maintaining a healthy weight for gout. In addition to clear information about the benefits of ULT [46], patient information resources should balance explanations of the potential for dietary modification both to avoid flare triggering and to lower urate, and also highlight the importance of weight loss. However, further evidence of the effect of dietary interventions, including weight loss, on both flare frequency and serum urate concentrations is needed.

Terms such as uric acid/urate, purines and acid/acidic were frequently conflated, leading to unnecessary dietary changes, such as avoidance of acidic foods. Expert consensus to standardize terminology might influence language used over time [38], but labels such as uric acid remain prevalent on gout websites and forums.

This study highlights the diversity and complexity of patients’ perceptions and experiences of the role of diet in managing gout. The sample is large for a qualitative study and includes people from different social backgrounds and age groups. However, participants were mostly White, and it would be useful to explore this topic with people from a wider range of ethnic backgrounds, because cultural factors might affect people’s dietary choices. The study draws on secondary analysis of data from two studies. Data collection and analysis were not simultaneous; therefore, modification of interview questions or additional probing in response to emerging findings about diet was not possible. However, as a result, participants were not recruited on the basis of having expressed an interest in gout and diet, but instead all shared their experiences and thoughts on this topic during conversations about living with gout more generally.

### Conclusions

We found that patients with gout commonly initiate dietary changes as a self-management strategy, providing greater understanding of the reasons why patients attempt management of gout through diet, in addition to the types of changes they make. We have identified opportunities for intervention and education about the dietary changes that might be beneficial, and to increase uptake and adherence to ULT: greater emphasis in patient educational materials on the importance of lowering urate; improving patient access to evidence-based professional advice about diet; maximizing opportunities to reduce/prevent flares through ULT, removing desperation and desire for control as drivers for dietary experimentation. Addressing reasons why patients explore dietary solutions and drawing on strong evidence to communicate clearly will both be crucial factors in improving long-term clinical management and patient experience.

## Data Availability

Data are available upon reasonable request. Data can be accessed via the Keele University data repository at: medicine.datasharing@keele.ac.uk
